# Egr2 and 3 control adaptive immune responses by temporally uncoupling expansion from T cell differentiation

**DOI:** 10.1084/jem.20160553

**Published:** 2017-06-05

**Authors:** Tizong Miao, Alistair L.J. Symonds, Randeep Singh, Janine D. Symonds, Ane Ogbe, Becky Omodho, Bo Zhu, Suling Li, Ping Wang

**Affiliations:** 1 The Blizard Institute, Barts and The London School of Medicine and Dentistry, Queen Mary University of London, London E1 2AT, England, UK; 2 Bioscience, Brunel University, Uxbridge UB8 3PH, England, UK; 3 Centre for Mathematics and Physics in the Life Sciences and Experimental Biology (CoMPLEX), University College London, London WC1E 6BT, England, UK; 4 Institute of Cancer, Xinqiao Hospital, Third Military Medical University, Chongqing 400037, People’s Republic of China

## Abstract

Egr2 and 3 are important for maintaining immune homeostasis. Here we define a fundamental function of Egr2 and 3 operating as a checkpoint that controls the transition between clonal expansion and differentiation of effector T cells. Egr2 and 3 deficiency resulted in defective clonal expansion but hyperactivation and excessive differentiation of T cells in response to viral infection. Conversely, sustained Egr2 expression enhanced expansion but severely impaired effector differentiation. Egr2 bound to and controlled the expression of genes regulating proliferation (Myc and Myb) and differentiation repressors (Bcl6, Id3), while repressing transcription factors required for effector function (Zeb2, RORa, RORc, and Bhlhe40). Egr2 and 3 expression in T cells was regulated reciprocally by antigen and IFNγ, providing a mechanism for adjusting proliferation and differentiation of individual T cells. Thus, Egr2 and 3 are upstream regulators of effector CD4 and CD8 T cells that are essential for optimal responses with limited immunopathology.

## Abstract (precis)

Miao et al. report a checkpoint mediated by Egr2 and 3 that controls the transition between T cell clonal expansion and differentiation by regulating genes involved in proliferation and differentiation, which is essential for optimal immune responses with limited immunopathology.

## Introduction

T cell clonal expansion and differentiation to effector cells are the hallmarks of adaptive immune responses (Kaech et al., 2002; Williams and Bevan, 2007). Although the initiation of clonal expansion and differentiation results in fundamental changes in the cellular function of naive T cells, in the course of responses to infection, activated T cells display extraordinary diversification in proliferation, differentiation, and the development of memory cells resulting from changes in external signals in the microenvironment such as antigens, inflammation, and co-stimulation (Buchholz et al., 2013; Gerlach et al., 2013). The adjustment of individual T cells in response to changes in external signals is important to achieve a robust response while controlling immunopathology (Buchholz et al., 2013; Gerlach et al., 2013). TCR signaling is required for activation and cell cycle progression leading to rapid clonal expansion (Kaech et al., 2002; Williams and Bevan, 2007). However, functional differentiation is induced by a combination of signals including antigens, inflammatory conditions, and cytokines, which induce differentiation programs regulated by transcription factors such as T-bet, Eomes, Runx2, Runx3, Id2, Id3, and BLIMP-1, leading to the acquisition of specific functions including cytotoxicity for CD8 T cells and Th function for CD4 cells, and also to form memory T cells (Kaech and Cui, 2012). Most of the known regulators important for adaptive responses of T cells affect both clonal expansion and effector differentiation (Kaech and Cui, 2012). Therefore, clonal expansion is considered to be coupled with effector differentiation and the development of memory. Findings from individual transcription factors involved in effector differentiation such as T-bet demonstrate that clonal expansion and differentiation are regulated by transcriptional networks rather than by individual transcription factors (Intlekofer et al., 2005). In addition, the diversity in clonal expansion and differentiation of individual T cells carrying the same TCR (Buchholz et al., 2013; Gerlach et al., 2013) indicates that there may be upstream regulators controlling clonal expansion and differentiation based on signals encountered in the microenvironment during adaptive immune responses.

Egr2 and 3 are closely related members of the Egr zinc finger transcription factor family with important roles in controlling the self-tolerance of lymphocytes and the development of NKT cells (Harris et al., 2004; Safford et al., 2005; Anderson et al., 2006; Lazarevic et al., 2009). Egr2 and 3 are induced in both naive and tolerant T cells (Harris et al., 2004; Safford et al., 2005; Anderson et al., 2006). The importance of Egr2 and 3 in controlling the development of autoimmunity was discovered in aged CD2-specific Egr2-deficient mice and in CD2-specific Egr2- and Egr3-deficient mice (Zhu et al., 2008; Li et al., 2012). Interestingly, despite increased homeostatic proliferation, and in contrast to findings from Egr2-transfected T cell lines (Safford et al., 2005), Egr2 or 3 single-deficient T cells respond normally to TCR stimulation in vitro (Zhu et al., 2008; Li et al., 2012), whereas proliferation of Egr2- and Egr3-deficient T cells is impaired (Li et al., 2012). Egr2 and 3 are highly induced in naive T cells at the early stages of responses to infection and antigen stimulation in vivo (Anderson et al., 2006; Best et al., 2013), suggesting that Egr2 and 3 may regulate T cell–mediated adaptive immune responses. Recently, Egr2 was found to be important for differentiation of T cells in response to viral infection by directly binding to the *Tbx21* locus and promoting the expression of T-bet (Du et al., 2014). However, defective responses to viral infection were not observed in a similar model from another study (Ramón et al., 2010).

Here, we discovered a fundamental and overlapping function of Egr2 and 3 for temporally uncoupling clonal expansion from differentiation of viral responding T cells. T cells that lack Egr2 and 3 were severely impaired in expansion but displayed excessive inflammatory responses, resulting in severe inflammatory pathology but poor viral clearance. In contrast, forced expression of Egr2 significantly increased clonal expansion, but the expanded T cells failed to differentiate. Egr2 and 3 were rapidly induced in naive T cells by antigen and promptly inhibited by IFNγ through an inhibitory feedback mechanism, which allows optimal clonal expansion and coupling of expansion with effector differentiation. Egr2 and 3 directly promoted expression of proliferation regulators (Myc and Myb) and differentiation repressors (Bcl6 and Id3) but suppressed transcription factors involved in effector differentiation (Zeb2, Bhlhe40, RORa, and RORc). Thus, temporally regulated expression of Egr2 and 3 induced in naive T cells in response to virus infection is essential for optimal adaptive immune responses with minimum pathology.

## Results

### Egr2 and 3 reciprocally control the activation and clonal expansion of T cells

**Figure 1.**
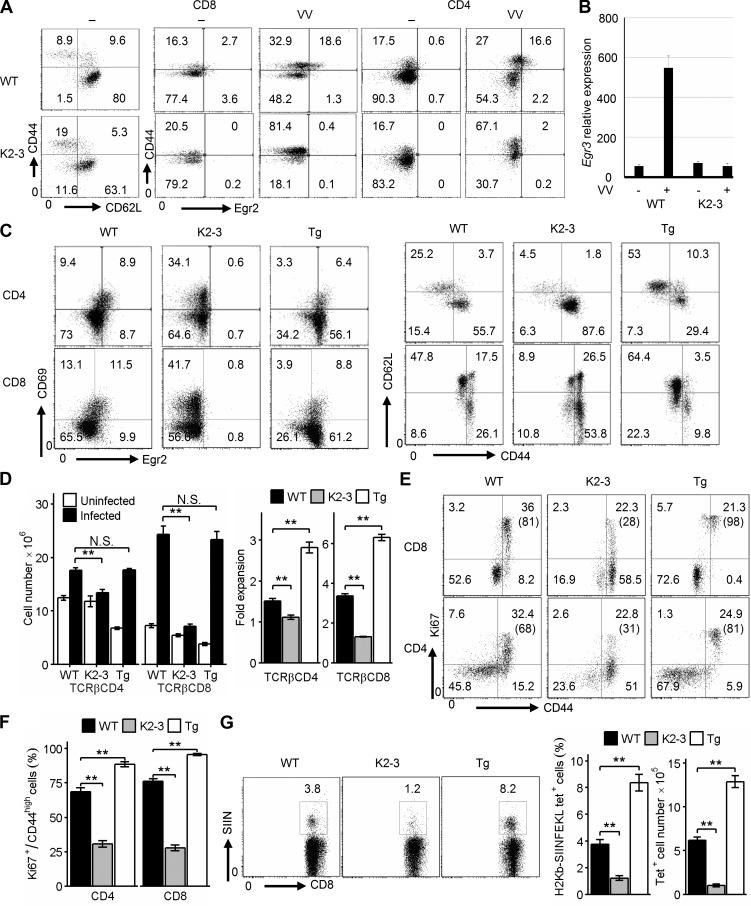
**Egr2 and 3 suppress activation but are required for clonal expansion of T cells.** WT and CD2-Egr2/3^−/−^ (K2-3) mice were infected with OVA-VV_WR_ i.n. (A and B) 7 d after infection, CD4 and CD8 T cells from spleen and lymph nodes were analyzed for CD62L and CD44 or CD44 and Egr2 expression (A) and Egr3 expression (B) by RT-PCR. (C) Activation phenotypes of CD4 and CD8 T cells from WT, K2-3, and CD2-Egr2 transgenic (Tg) mice. (D) T cell numbers before and after viral infection in WT, K2-3, and Tg mice (left) and fold expansion after infection (right). (E and F) Cells from spleen and lymph nodes of infected WT, K2-3, and Tg mice were analyzed for Ki67 and CD44 expression on gated CD4 and CD8 cells, with the percentages of Ki67^+^ cells among the CD44^high^ population indicated in parentheses (E); and the percentages of Ki67^+^ cells among CD44^high^ cells were analyzed (F). (G) Kb-SIINFEKL tetramer–positive CD8 cells from spleen and lymph nodes of WT, K2-3, and Tg mice 7 d after infection. Data in A–G are from pooled cells of five mice of each genotype and are representative of three independent experiments. Data in B are means ± SD, and data in D, F, and G are means ± SEM and were analyzed with Kruskal–Wallis tests, followed by Conover tests with Benjamini–Hochberg correction. N.S., not significant; **, P < 0.01.

Egr2 and 3 are induced in naive T cells by infection or antigen stimulation in vivo (Anderson et al., 2006; Best et al., 2013). To investigate the role of Egr2 and 3 in T cells in antiviral responses, we infected WT, CD2-Egr2 transgenic and CD2-Egr2/3^−/−^ mice aged between 7 and 8 wk i.n. with vaccinia virus encoding OVA (OVA-VV_WR_). After backcrossing more than 30 times to the C57BL/6 background, T cells from 8-wk-old CD2-Egr2/3^−/−^ mice showed levels of effector phenotype T cells similar to those in WT mice (Fig. 1 A). Therefore, we used young mice (7–8 wk) that did not show signs of autoimmunity throughout this study. Egr2 and 3 were barely detectable in T cells from naive WT mice (Fig. 1, A and B). 7 d after infection with OVA-VV_WR_, Egr2 was induced in CD44^high^ T cells (Fig. 1 A). Induction of Egr3 was also detected in CD8 T cells from infected WT mice (Fig. 1 B). The induction of Egr2 expression in activated CD4 and CD8 T cells was further demonstrated by coexpression of CD69 with Egr2 in CD4 and CD8 T cells from virus-infected WT mice (Fig. 1 C). Although ∼40% of CD8 T cells in WT mice were CD44^high^, the proportion of CD44^high^ cells was more than 75% in CD2-Egr2/3^−/−^ mice but less than 15% in CD2-Egr2 transgenic mice (Fig. 1 C). Similar expression patterns were seen for CD69 and CD44 in CD4 T cells (Fig. 1 C), indicating that Egr2 and 3 are not required for but control T cell activation. Although the numbers of T cells in naive CD2-Egr2 transgenic and CD2-Egr2/3^−/−^ mice were lower than in WT mice (Li et al., 2011, 2012), the expansion of T cells in infected CD2-Egr2/3^−/−^ mice was severely defective, whereas expansion was enhanced in CD2-Egr2 transgenic mice (Fig. 1 D). Although ∼70% of CD44^high^ T cells in WT mice expressed Ki67, less than a third of CD44^high^ T cells in CD2-Egr2/3^−/−^ mice expressed this proliferation marker (Fig. 1, E and F). Interestingly, despite the decreased proportion of CD44^high^ T cells in CD2-Egr2 transgenic mice, ∼90% of this population expressed Ki67 (Fig. 1, E and F). Consistent with this differential proliferation, the number of Kb-SIINFEKL–specific CD8 T cells in infected CD2-Egr2/3^−/−^ mice was only ∼10^5^ compared with ∼12 × 10^5^ in CD2-Egr2 transgenic mice and ∼6 × 10^5^ in WT mice (Fig. 1 G). Thus, Egr2 and 3, induced in activated T cells, control T cell activation but also promote clonal expansion of virus-specific T cells.

### Egr2 and 3 suppress differentiation of effector T cells

**Figure 2.**
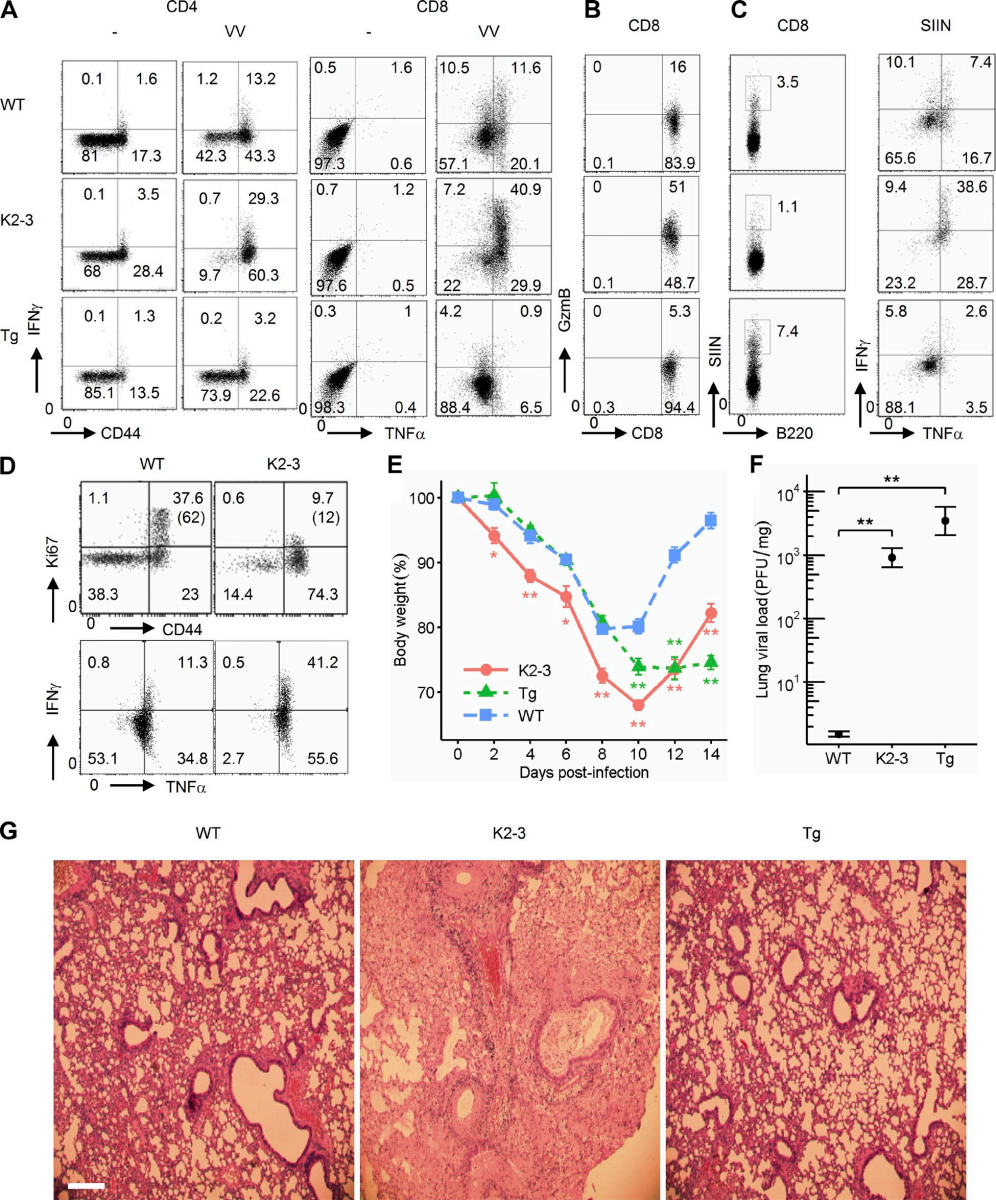
**Egr2 and 3 control differentiation of effector T cells.** WT, CD2-Egr2/3^−/−^ (K2-3), and CD2-Egr2 transgenic (Tg) mice were infected with OVA-VV_WR_ i.n. and analyzed 7 d after infection. (A) CD4 and CD8 cells from spleen and lymph nodes of uninfected and infected mice were incubated with virus-infected LB27.4 cells for 16 h before analysis of IFNγ- and TNF-producing cells. (B) Granzyme B–producing CD8 cells from spleen and lymph nodes of infected mice were analyzed after in vitro stimulation as in A. (C) CD8 cells were isolated from spleens of infected mice and incubated with virus-infected LB27.4 cells for 16 h in vitro. CD8^+^Kb-SIINFEKL-tetramer^+^ cells (left) were analyzed for IFNγ and TNF production (right). (D) CD8 T cells among lung-infiltrating lymphocytes from infected mice were gated for analysis of proliferation and production of cytokines. The percentages of Ki67^+^ cells among the CD44^high^ population are indicated in parentheses. (E) Body weight loss of infected mice. (F) Viral titer in lung tissue specimens. (G) H&E staining of lung tissues 7 d after infection. Bar, 100 µm. Data in A–D are from pooled cells from five mice of each genotype and are representative of three independent experiments. Data in E–G are representative of five independent experiments with four to five mice per group. Data in E and F are means ± SEM and were analyzed with Kruskal–Wallis tests, followed by Conover tests with Benjamini–Hochberg correction. *, P < 0.05; **, P < 0.01.

Clonal expansion is suggested to be coupled with effector differentiation in T cell adaptive immune responses (Kaech et al., 2002; Williams and Bevan, 2007). We have now shown that Egr2 and 3 are required for clonal expansion. Therefore, we assessed the differentiation of effector CD4 and CD8 T cells in response to viral infection by analysis of production of effector cytokines and granzyme B. We discovered that Egr2 and 3 promoted clonal expansion but profoundly suppressed differentiation of effector CD4 and 8 T cells. Although their clonal expansion was severely impaired, Egr2- and Egr3-deficient CD4 and CD8 T cells were hyperdifferentiated during antiviral responses, as indicated by the percentages of IFNγ-producing CD4 cells and IFNγ-, TNF-, and granzyme Β–producing CD8 T cells, which were more than double those in WT counterparts (Fig. 2, A–C). In contrast, the expression of effector cytokines by CD4 and CD8 T cells in infected CD2-Egr2 transgenic mice was less than a third of that from WT counterparts (Fig. 2, A–C), indicating that clonal expansion was largely uncoupled from effector differentiation in infected CD2-Egr2 transgenic mice. We also found that lung CD8 cells from infected CD2-Egr2/3^−/−^ mice had decreased Ki67 expression but increased IFNγ and TNF expression compared with lung CD8 cells from infected WT mice (Fig. 2 D) consistent with the results from lymphoid organs. These findings demonstrate a novel and fundamental function of Egr2 and 3 for promoting expansion while inhibiting differentiation, and indicate that Egr2 and 3 function has to be regulated for optimal coupling of clonal expansion with differentiation of effector cells.

### Controlled expression of Egr2 and 3 in T cells is essential for antiviral responses

Although CD2-Egr2/3^−/−^ and CD2-Egr2 mice had opposite patterns of clonal expansion and differentiation, both lines had more severe infection than WT mice (Fig. 2 E) with impaired viral clearance (Fig. 2 F). However, the immunopathology in infected lungs was altered distinctively in CD2-Egr2/3^−/−^ and CD2-Egr2 transgenic mice. CD2-Egr2/3^−/−^ mice had severe inflammatory pathology in the lungs after i.n. infection with OVA-VV_WR_ (Fig. 2 G), whereas lung inflammation and mononucleocyte infiltration in infected lungs were much milder in CD2-Egr2 transgenic mice compared with WT counterparts (Fig. 2 G). The reciprocally altered lung immunopathology mirrored the reciprocal alterations in T cell activation and differentiation in these mice, demonstrating that dysregulated expression of Egr2 and 3 in T cells results in impaired adaptive immune responses.

### Overlapping role of Egr2 and 3 in the control of T cell responses

**Figure 3.**
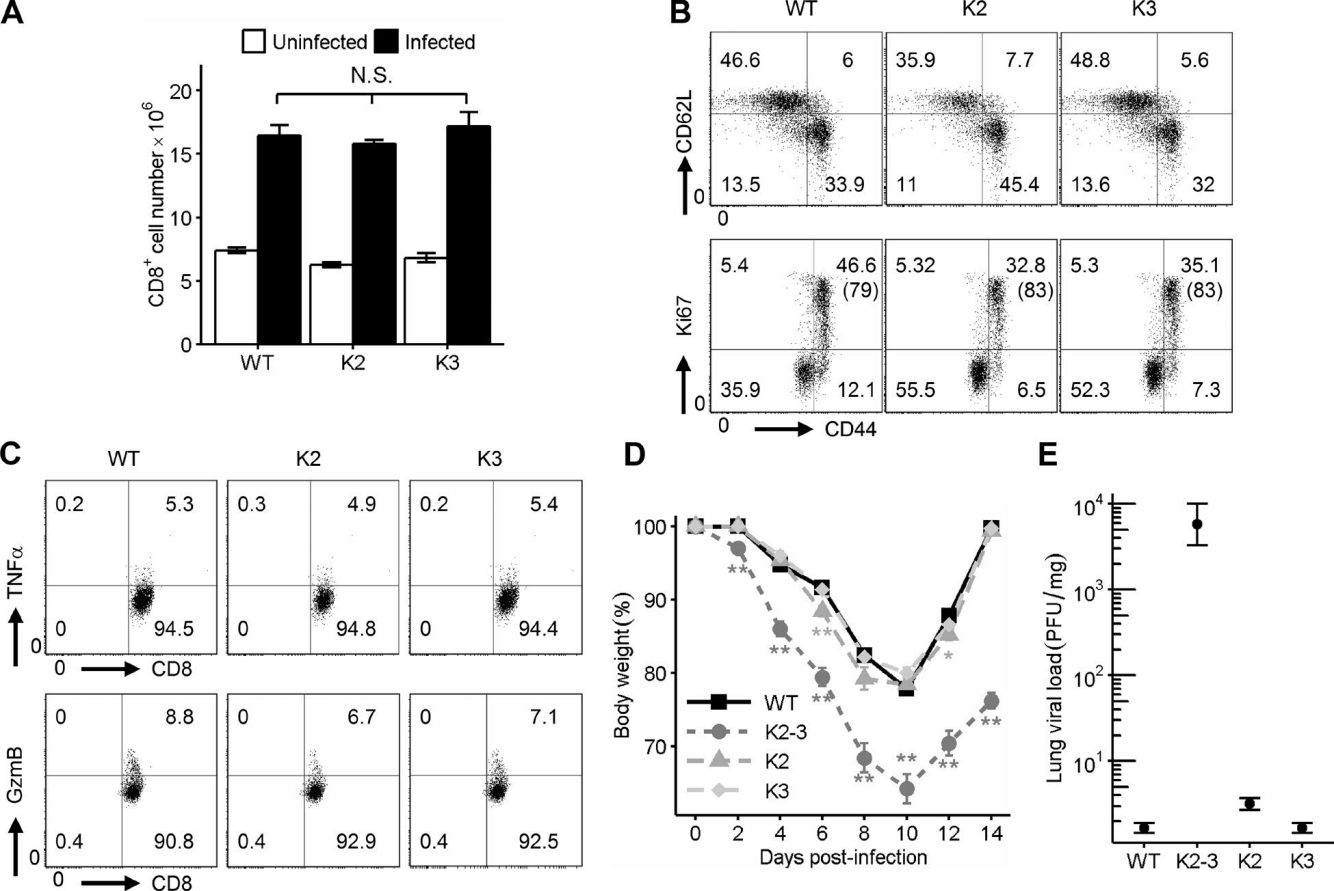
**Overlapping function of Egr2 and 3 in control of T cell proliferation and differentiation.** WT, CD2-Egr2 knockout (K2), and Egr3 knockout (K3) mice were infected with OVA-VV_WR_ i.n. for 7 d. (A–C) CD8 T cells from spleen and lymph nodes were analyzed for expansion (A), activation and proliferation (B), and production of cytokines (C). The percentages of Ki67^+^ cells among the CD44^high^ population are indicated in parentheses in B. (D and E) Body weight loss (D) and lung viral load (E) were measured compared with CD2-Egr2/3^−/−^ (K2-3) mice. A–E are representative of two independent experiments with four mice per group. Data in A, D, and E are means ± SEM and were analyzed with Kruskal–Wallis tests, followed by Conover tests with Benjamini–Hochberg correction. N.S., not significant; *, P < 0.05, **, P < 0.01.

To determine whether the altered responses of CD2-Egr2/3^−/−^ mice are caused by deficiency in Egr2, Egr3, or both, we analyzed mice with a single defect in either Egr2 or Egr3 in T cells. T cells with a single defect in either Egr2 or Egr3 did not have significant changes in expansion, proliferation, activation markers, or differentiation in response to viral infection (Fig. 3, A–C). Furthermore, mice with a single defect in either Egr2 or Egr3 in T cells did not have impaired responses to viral infection (Fig. 3, D and E). Collectively, these results demonstrate that the altered responses of CD2-Egr2/3^−/−^ mice are caused by deficiency in both Egr2 and Egr3, indicating an overlapping function of Egr2 and 3 in the control of T cell responses.

### The function of Egr2 and 3 is T cell intrinsic

**Figure 4.**
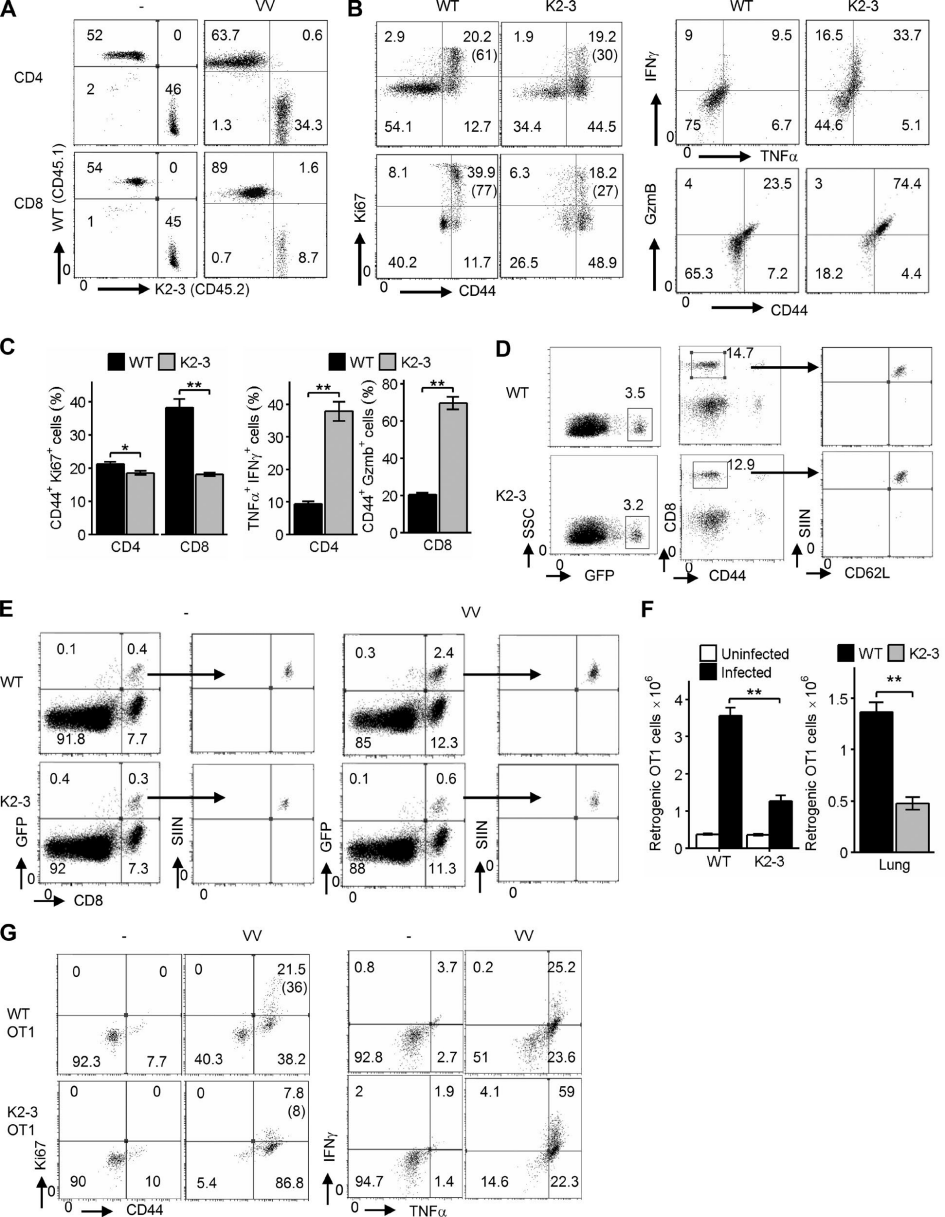
**Egr2 and 3 function is cell intrinsic.** (A–C) Irradiated WT mice were adoptively transferred with an equal number of BM cells from WT and CD2-Egr2/3^−/−^ (K2-3) mice. 8 wk after transfer, mice were infected with OVA-VV_WR_ and analyzed 7 d after infection. (A) Splenic cells from chimeric mice were stained with CD45.1, CD45.2, CD4, and CD8, and the proportion of WT (CD45.1) and K2-3 (CD45.2) CD4 and CD8 cells was determined by flow cytometry. (B and C) Gated WT (CD45.1) and K2-3 (CD45.2) CD4 and CD8 cells were analyzed for expression of the activation marker CD44 and the proliferation marker Ki67 (left) and TNF and IFNγ for CD4 cells and granzyme B for CD8 cells (right). The percentages of Ki67^+^ cells among the CD44^high^ population are indicated in parentheses in B. (D–G) WT and K2-3 OT1 retrogenic T cells were analyzed in recipient mice before and after infection. (D) GFP^+^CD8^+^CD44^lo^ cells were isolated from WT and K2-3 OT1 retrogenic mice (left and middle) and confirmed as CD62L^+^Kb-SIINFEKL-tetramer^+^ cells (right). 3 × 10^5^ to 5 × 10^5^ WT or Egr2/3^−/−^ retrogenic-OT1 cells were adoptively transferred to separate naive WT mice. 1 d after transfer, mice were infected with OVA-VV_WR_ and analyzed 7 d after infection. (E and F) Retrogenic-OT1 GFP^+^CD8^+^Kb-SIINFEKL-tetramer^+^ cells among spleen and lymph node cells from recipient mice were identified (E), and the numbers of WT and K2-3 retrogenic-OT1 cells in spleen and lymph nodes (left) and lung (right) were quantified (F). (G) Expression of proliferation and activation markers and production of effector cytokines by WT-retrogenic-OT1 and K2-3–retrogenic-OT1 cells gated as in E. The percentages of Ki67^+^ cells among the CD44^high^ population are indicated in parentheses. Data are from pooled cells from four mice of each genotype and are representative of three (A–C) or two (D–G) independent experiments. Data in C and F are means ± SEM and were analyzed with Mann–Whitney two-tailed tests. *, P < 0.05; **, P < 0.01.

To assess whether the function of Egr2 and 3 during antiviral responses is T cell intrinsic, we generated chimeras by reconstitution of irradiated WT mice with an equal number of BM cells from WT and CD2-Egr2/3^−/−^ mice. Consistent with CD2-Egr2/3^−/−^ mice, Egr2- and Egr3-deficient T cells in the chimeras displayed impaired expansion and enhanced differentiation in response to viral infection (Fig. 4, A–C). However, T cells from WT BM showed normal responses in terms of expansion and differentiation (Fig. 4, A–C). To exclude the influence of developmental defects (Li et al., 2011, 2012) on the function of Egr2/3 deficient T cells, and to analyze antigen-specific responses, we generated OT1 retrogenic mice by reconstituting irradiated C57BL/6 mice with BM cells from either WT or CD2-Egr2/3^−/−^ mice after transduction with an OT1 TCRα and β retroviral construct, which also carries a GFP marker (Holst et al., 2006a,b). Naive WT and Egr2/3^−/−^ retrogenic OT1 cells were isolated and adoptively transferred to separate naive WT mice. GFP^+^CD8^+^Kb-SIINFEKL-tetramer^+^ retrogenic OT1 cells were analyzed before and after infection in recipient mice. Consistent with the results from CD2-Egr2/3^−/−^ mice, Egr2/3-deficient retrogenic OT1 cells had severe defects in proliferation but were highly activated and produced excessive levels of effector cytokines compared with WT retrogenic-OT1 cells in response to OVA-VV_WR_ infection (Fig. 4, D–G). These results demonstrate that the reciprocal defects in clonal expansion and differentiation of Egr2- and Egr3-deficient T cells in adaptive immune responses are cell intrinsic and not a result of developmental defects.

**Figure 5.**
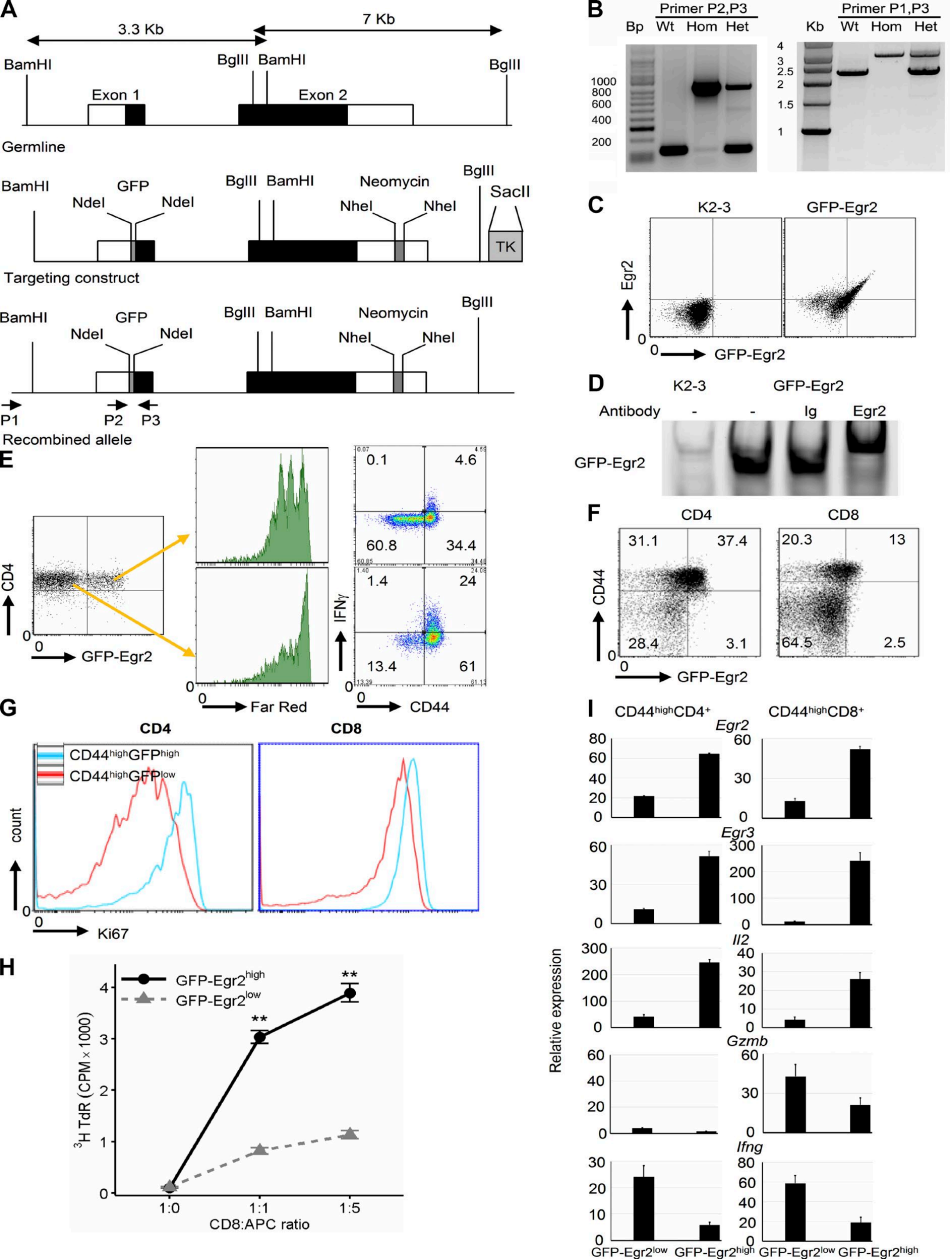
**Expression of Egr2 and 3 control the proliferation and differentiation of activated T cells in response to viral infection.** (A) Generation of the GFP-Egr2 knock-in construct resulting in GFP fusion to the N terminus of Egr2. (B) PCR screening of homologous recombination with primers indicated in A. (C and D) CD4 T cells from spleen and lymph nodes of GFP-Egr2 knock-in mice (GFP-Egr2) and CD2-Egr2/3^−/−^ (K2-3) mice were stimulated for 16 h with anti-CD3 and anti-CD28 in vitro and then analyzed for GFP-Egr2 expression by GFP and Egr2 staining (C) and for interaction of GFP-Egr2 with its consensus DNA binding sequence by EMSA (D). (E) CD4 T cells from K2-3 mice were transduced with a GFP-Egr2-lentiviral expression construct. GFP-Egr2–positive and –negative cells were sorted and labeled with CellTrace Far-Red before stimulation with anti-CD3 and anti-CD28 in vitro for analysis of proliferation and production of IFNγ. (F–I) GFP-Egr2 knock-in mice were infected with OVA-VV_WR_ i.n. and analyzed 7 d after infection. Splenic CD4 and CD8 cells were analyzed for GFP-Egr2 and CD44 expression (F), and gated CD44^high^GFP-Egr2^high^ and CD44^high^GFP-Egr2^low^ CD4 and CD8 cells were assessed for Ki67 expression (G). (H) CD44^high^GFP-Egr2^high^ and CD44^high^GFP-Egr2^low^ CD8 cells were isolated by cell sorting and incubated with OVA-VV_WR_–infected LB27.4 cells as antigen-presenting cells. Proliferation was measured after 72 h. (I) Expression of the indicated transcripts in isolated CD44^high^GFP-Egr2^high^ and CD44^high^GFP-Egr2^low^ T cells. C–E are representative of three independent experiments. Data in F–I are from pooled cells from four mice of each genotype and are representative of three independent experiments. Data in I are means ± SD. Data in H are means ± SEM and were analyzed with a Mann–Whitney two-tailed test. **, P < 0.01.

To further assess the intrinsic function of Egr2 and 3 in T cells during antiviral responses, we created GFP-Egr2 knock-in mice (Fig. 5, A–C). GFP was fused to the N terminus of Egr2, which does not affect the interaction of Egr2 with its target DNA (Fig. 5 D) or Egr2 function, as indicated by restored function of Egr2- and Egr3-deficient T cells after transduction with lentivirus carrying the GFP-Egr2 fusion gene (Fig. 5 E). Consistent with WT mice, high levels of GFP-Egr2 were detected in CD44^high^ T cells in response to viral infection (Fig. 5 F). 7 d after infection, CD44^high^GFP-Egr2^high^ and CD44^high^GFP-Egr2^low^ cells were assessed for their proliferation and effector function. CD44^high^GFP-Egr2^high^ and CD44^high^GFP-Egr2^low^ cells displayed asymmetric responses in terms of proliferation and differentiation, resembling the results from CD2-Egr2 transgenic and CD2-Egr2/3^−/−^ mice, respectively. Ki67 was highly expressed in CD44^high^GFP-Egr2^high^ but was low in CD44^high^GFP-Egr2^low^ cells (Fig. 5 G). CD8^+^CD44^high^GFP-Egr2^high^ cells underwent strong proliferation, whereas CD8^+^CD44^high^GFP-Egr2^low^ cells proliferated poorly in response to viral antigen stimulation in vitro (Fig. 5 H). In response to virus infection, CD44^high^GFP-Egr2^high^ and CD44^high^GFP-Egr2^low^ T cells produced IL2 and effector cytokines differentially, again similar to T cells from CD2-Egr2 transgenic and CD2-Egr2/3^−/−^ mice. CD44^high^GFP-Egr2^high^ T cells expressed high levels of IL2 but very low IFNγ, whereas CD44^high^GFP-Egr2^low^ cells expressed low levels of IL2 but high levels of IFNγ (Fig. 5 I). Similar to the differential production of IFNγ, CD8^+^CD44^high^GFP-Egr2^high^ cells expressed low levels of granzyme B, whereas CD8^+^CD44^high^GFP-Egr2^low^ cells expressed high levels of granzyme B (Fig. 5 I). Egr3 expression was higher in CD44^high^GFP-Egr2^high^ cells than CD44^high^GFP-Egr2^low^ cells (Fig. 5 I), supporting the notion of an overlapping function between these two transcription factors. These results demonstrate that Egr2 and 3 play an intrinsic role in controlling clonal expansion and differentiation of T cells and confirm that the dysregulated functions of T cells resulting from Egr2 and 3 deficiency or sustained expression of Egr2 are not caused by developmental defects.

### Egr2 and 3 reciprocally regulate genes involved in proliferation and effector differentiation

**Figure 6.**
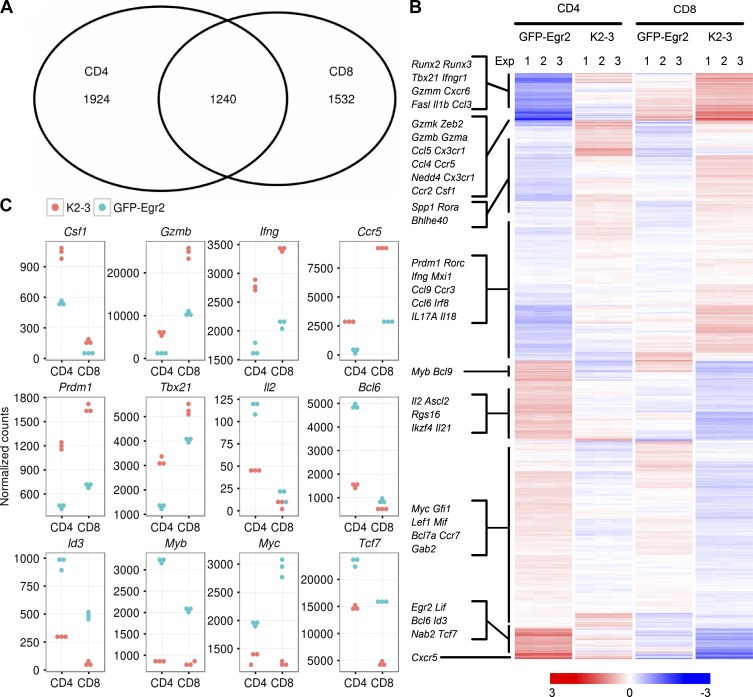
**Egr2 and 3 reciprocally regulate genes involved in proliferation and differentiation.** Genome-wide mRNA expression profiling by RNA-seq was performed on CD44^high^GFP-Egr2^high^CD4^+^ and CD44^high^GFP-Egr2^high^CD8^+^ cells from GFP-Egr2 knock-in mice and CD44^high^CD4^+^ and CD44^high^CD8^+^ cells from CD2-Egr2/3^−/−^ mice at day 7 after infection with VV_WR_. Differentially expressed genes were defined as ≥1.5-fold difference between the two groups with a false discovery rate of <0.05. (A) Genes differentially expressed between Egr2/3^−/−^ and Egr2^high^ CD4 and CD8 T cells were compared. (B) Heat map shows the mRNA expression of genes differentially expressed between GFP-Egr2 and Egr2/3^−/−^ cells, in both CD4 and CD8 populations, with the individual experiments (Exp) and selected biologically relevant genes indicated. (C) Expression of selected genes in CD4 and CD8 cells. The RNA-seq data are from three biological replicates, each with three to five mice, for each group.

To investigate the genes regulated by Egr2 and 3 in Egr2-expressing T cells in infected mice in vivo, CD44^high^GFP-Egr2^high^ CD4 and CD8 cells from GFP-Egr2 mice and CD44^high^ CD4 and CD8 T cells from CD2-Egr2/3^−/−^ mice were isolated after viral infection and analyzed by RNA-seq. The expression profiles from three independent infection experiments showed that more than a third of genes dysregulated in CD4 cells were also altered in CD8 T cells and vice versa (Fig. 6 A). The genes up-regulated in Egr2- and Egr3-deficient T cells were mostly associated with effector differentiation and function of effector T cells (Table 1, Fig. 6 B, and Dataset S1), including lineage-specific transcription factors (*Tbx21*, *Runx2*, *Runx3*, *Rora*, *Rorc*, *Bhlhe40*, *Zeb2*, and *Prdm1*; Kaech and Cui, 2012); genes required for effector function (*Ifng*, *Gzma*, *Gzmb*, *Gzmk*, *Csf1*, *Il17a*, *Il1b*, and *Il18*; Kaech et al., 2002; Williams and Bevan, 2007) and migration of effector T cells (*Ccl4*, *Ccr2*, *Ccr3*, and *Ccr5*; Luther and Cyster, 2001); and inhibitors of proliferation (*Mxi1* and *Mxd1*; Fig. 6 B; Ayer et al., 1993; Zervos et al., 1993). In contrast, the down-regulated genes included key genes required for T cell expansion (*Il2*, *Myb*, *Myc*, and *Bcl9*; Fig. 6 B; Lieu et al., 2004; Mani et al., 2009; Wang et al., 2011; Nie et al., 2012). Interestingly, transcription factors known to be involved in T follicular helper (Tfh) cell development (*Ascl2*, *Bcl6*, *Tcf7*, *Lef1*; Nurieva et al., 2009; Yu et al., 2009; Liu et al., 2014; Choi et al., 2015; Xu et al., 2015) were down-regulated (Fig. 6 B), which is consistent with our previous findings that Tfh development is defective in Egr2- and Egr3-deficient CD4 T cells (Ogbe et al., 2015). In addition, repressors of effector differentiation such *Id3* and *Bcl6* were also down-regulated in Egr2- and Egr3-deficient T cells (Shaffer et al., 2000; Yang et al., 2011). Although most of the genes were altered in both CD4 and CD8 T cells from infected CD2-Egr2/3^−/−^ mice, some genes, including *Csf1* and *Bcl6*, were altered predominantly in CD4, whereas others, such as *Gzmb*, were mainly changed in CD8 cells (Fig. 6 C). The genes important in both CD4 and CD8 effector T cells, such as *Ifng*, *Ccr5*, *Prdm1*, *Tbx21*, *Id3*, *Myc*, and *Myb*, were up or down-regulated in both cell types (Fig. 6 C).

**Table 1.** Examples of differentially expressed genes in Egr2- and Egr3-deficient T cells with key functions in T cells

Altered expression	Cytokine	Effector function	Homing	Proliferation	Metabolism	Transcription factors
Down-regulated	*Il2*	*Bcl6*	*Cxcr5*	*Bcl6*	*Tpi1*	*Bcl6*
	*Il21*	*Tcf7*	*Ccr7*	*Bcl9*	*Pfkfb3*	*Tcf7*
	*Il6ra*	*Lef1*		*Bcl7a*	*Adk*	*Lef1*
	*Il27ra*	*Ascl2*		*Myc*	*Ak4*	*Ascl2*
		*Id3*		*Myb*	*Ak7*	*Id3*
					*Pgm2*	*Bcl9*
					*Ldhb*	*Bcl7a*
						*Myc*
						*Myb*
Up-regulated	*Il1b*	*Tbx21*	*Ccl8*	*Mxi1*	*Npc1*	*Tbx21*
	*Il17a*	*Zeb2*	*Cx3cr1*	*Mxd1*	*Upp1*	*Zeb2*
	*Il23r*	*Rora*	*Ccl6*		*Crot*	*Rora*
	*Tnfrsf13c*	*Rorc*	*Ccl3*		*Xdh*	*Rorc*
	*Il18*	*Rara*	*Ccl5*			*Rara*
	*Il7r*	*Runx2*	*Ccr2*			*Runx2*
	*Csf1*	*Runx3*	*Ccl9*			*Runx3*
	*Ifng*	*Bhlhe40*	*Ccr6*			*Bhlhe40*
	*Il10*	*Ifng*	*Cxcr6*			*Prdm1*
		*Gzmb*	*Ccl4*			*Mxi1*
		*Gzmk*	*Ccr3*			*Mxd1*
		*Gzma*	*Ccr1*			
		*Gzmc*	*Ccr5*			
		*Il17a*				
		*Prdm1*				

**Figure 7.**
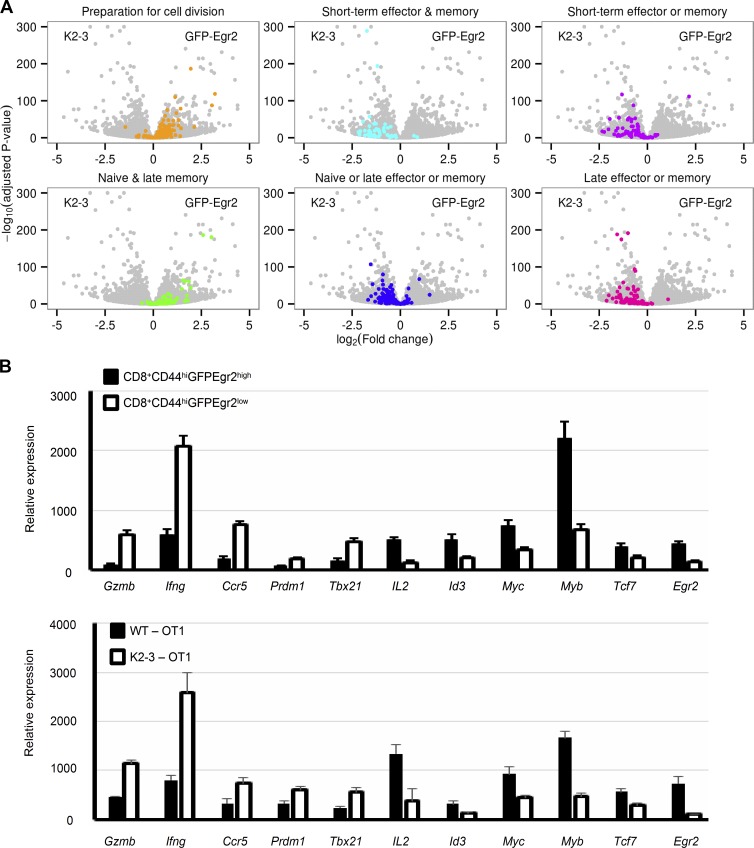
**Egr2 and 3 regulated genes are important for early proliferation of T cells in response to infection.** (A) Volcano plots of the entire RNA-seq dataset (gray) were overlaid with the indicated functional groups of genes (colors) previously defined in CD8 T cells at different stages of infection (Best et al., 2013), with positive and negative log2 fold changes indicating higher expression in GFP-Egr2 and CD2-Egr2/3^−/−^ (K2-3) cells, respectively. (B) Key genes differentially expressed between GFP-Egr2 and K2-3 cells in the RNA-seq dataset show similar patterns in both CD44^high^GFP-Egr2^high^CD8^+^ and CD44^high^GFP-Egr2^low^CD8^+^ cells from GFP-Egr2 knock-in mice (top) and CD8^+^Kb-SIINFEKL-tetramer^+^ cells from WT and CD2-Egr2/3^−/−^ mice (bottom) 7 d after infection with OVA-VV_WR_. Data are means ± SD and are representative of three independent experiments (each with *n* = 10 mice/group).

Recently, a kinetic study of the expression profiles of CD8 T cells from OT1 TCR transgenic mice during infection discovered distinct gene clusters that are associated with different stages and functions of the responding CD8 T cells (Best et al., 2013). We found that the genes down-regulated in Egr2- and Egr3-deficient T cells were predominantly among early-stage gene clusters with functions such as preparation for cell division and naive or late memory (Fig. 7 A), whereas up-regulated genes were mainly in later stages with functions in effector or effector and memory cells (Fig. 7 A). To further confirm the differential expression of Egr2/3-regulated genes, CD44^high^GFP-Egr2^high^ and CD44^high^GFP-Egr2^low^ CD8 cells from viral infected GFP-Egr2 knock-in mice and CD8^+^Kb-SIINFEKL-tetramer^+^ cells from viral infected WT and CD2-Egr2/3^−/−^ mice were analyzed for the expression of representative genes detected by RNA-seq. The expression of these genes in CD44^high^GFP-Egr2^low^ CD8 cells and Egr2/3^−/−^CD8^+^Kb-SIINFEKL-tetramer^+^ cells was consistent with their expression pattern in Egr2/3^−/−^ CD8 T cells (Fig. 7 B). Thus, the up- and down-regulated gene profiles were consistent with the defects in T cells observed in CD2-Egr2/3^−/−^ mice and the functions of Egr2^high^ and Egr2^low^ T cells from GFP-Egr2 knock-in mice in response to viral infection.

### Egr2 binds to and controls the expression of transcription factors important for clonal expansion and effector differentiation

**Figure 8.**
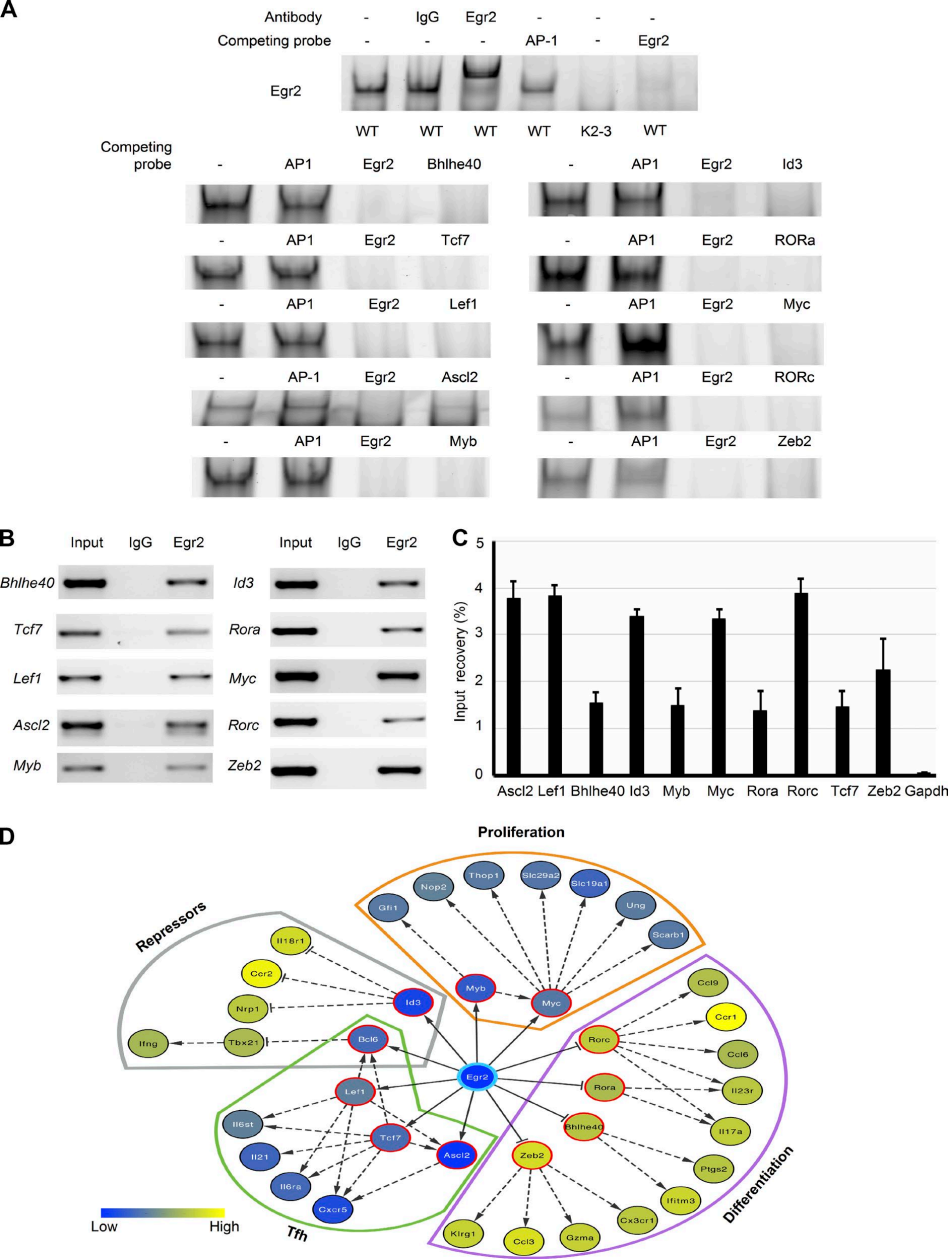
**Egr2 binds to and regulates the expression of transcription factors involved in the proliferation and differentiation of T cells.** (A) CD4 T cells from WT mice were stimulated for 16 h in vitro with anti-CD3 and anti-CD28 and nuclear proteins used for EMSA analysis. Probes derived from the potential Egr2-binding sites in the loci of the indicated transcription factors were used as competitors for the interaction of Egr2 with a labeled probe containing its consensus binding sequence. Unlabeled probe containing the AP-1 consensus sequence served as negative control, and unlabeled Egr2 consensus sequence served as positive control. The specificity of the band was confirmed by supershift. Nuclear proteins from stimulated CD2-Egr2/3^−/−^ (K2-3) CD4 T cells served as a further negative control. (B and C) ChIP assay of anti-Egr2 precipitates from anti-CD3 and anti-CD28 stimulated CD4 T cells from WT mice. Total chromatin extracts from the same cells were used as positive controls, and IgG precipitates served as negative controls. ChIP data are presented as the percentage recovery of input in C. (D) Model of the “Egr2-centric” gene expression network in T cells in response to viral infection. Nodes are colored according to the level of expression in K2-3 cells in the RNA-seq data (Fig. 6), and those with a red border are direct Egr2 target genes. A–C are representative of three independent experiments. Data in C are means ± SD.

The altered transcription profiles in Egr2- and Egr3-deficient T cells showed bias toward genes involved in expansion and effector function and included key transcription factors regulating proliferation and differentiation. Therefore, we analyzed the regulatory regions of these loci for potential binding sites for Egr2 using the programs Mulan and multiTF (Ovcharenko et al., 2005). Most loci had more than two potential binding sites in their regulatory regions. The potential binding sites were first assessed by electrophoretic mobility shift assay (EMSA) with probes derived from the potential binding sites as competitors for the interaction of Egr2 with its DNA consensus sequence (Ogbe et al., 2015). We first confirmed that Egr2 induced in naive CD4 T cells effectively bound to its DNA consensus sequence (Fig. 8 A). EMSA results showed that Egr2 from CD4 T cells of WT mice bound to most of the predicted sites (Fig. 8 A). The interactions were further validated by chromatin immunoprecipitation (ChIP) assays with primers flanking the binding sites (Fig. 8, B and C). In addition to direct interaction with the *Bcl6* locus (Ogbe et al., 2015), we found that Egr2 directly bound to the *Myc*, *Myb*, *Tcf7*, *Id3*, *Ascl2*, and *Lef1* loci, genes that were down-regulated in Egr2- and Egr3-deficient T cells, and also to the *Bhlhe40*, *Rora*, *Rorc*, and *Zeb2* loci, which were up-regulated in Egr2- and Egr3-deficient T cells in response to viral infection (Fig. 8, B and D). Consistent with the altered expression of these transcription factors, several of their target genes in Egr2- and Egr3-deficient T cells were also altered in the same fashion (Figs. 8 D and 6 B), indicating that Egr2 and 3 are upstream of specific transcription factors that are functionally important for proliferation and differentiation. Moreover, we confirmed binding of Egr2 to loci encoding transcription factors involved in Tfh development (*Ascl2*, *Lef1*, and *Tcf7*) that were down-regulated in Egr2- and Egr3-deficient CD4 T cells (Fig. 8, B and C), which extends our previous findings that Egr2 and 3 are important for Tfh development (Ogbe et al., 2015). *Bhlhe40*, *Rora*, *Rorc*, and *Zeb2* are transcription factors essential for differentiation of cytotoxic CD8 and Th1 and Th17 CD4 cells (Kaech and Cui, 2012; Lin et al., 2014; Dominguez et al., 2015; Omilusik et al., 2015), and we have shown that they are directly regulated by Egr2. Thus, Egr2 and 3 are upstream regulators of effector differentiation that promote Tfh cell differentiation while suppressing other effector functions. The “Egr2-centric” transcriptional network (Fig. 8 D and Table S1) highlights that Egr2 and/or 3 is an upstream regulator of early-stage adaptive responses by reciprocally regulating proliferation and Tfh cell differentiation versus differentiation of cytotoxic and effector Th cells.

### Expression of Egr2 and 3 in T cells is reciprocally regulated by antigen and IFNγ

**Figure 9.**
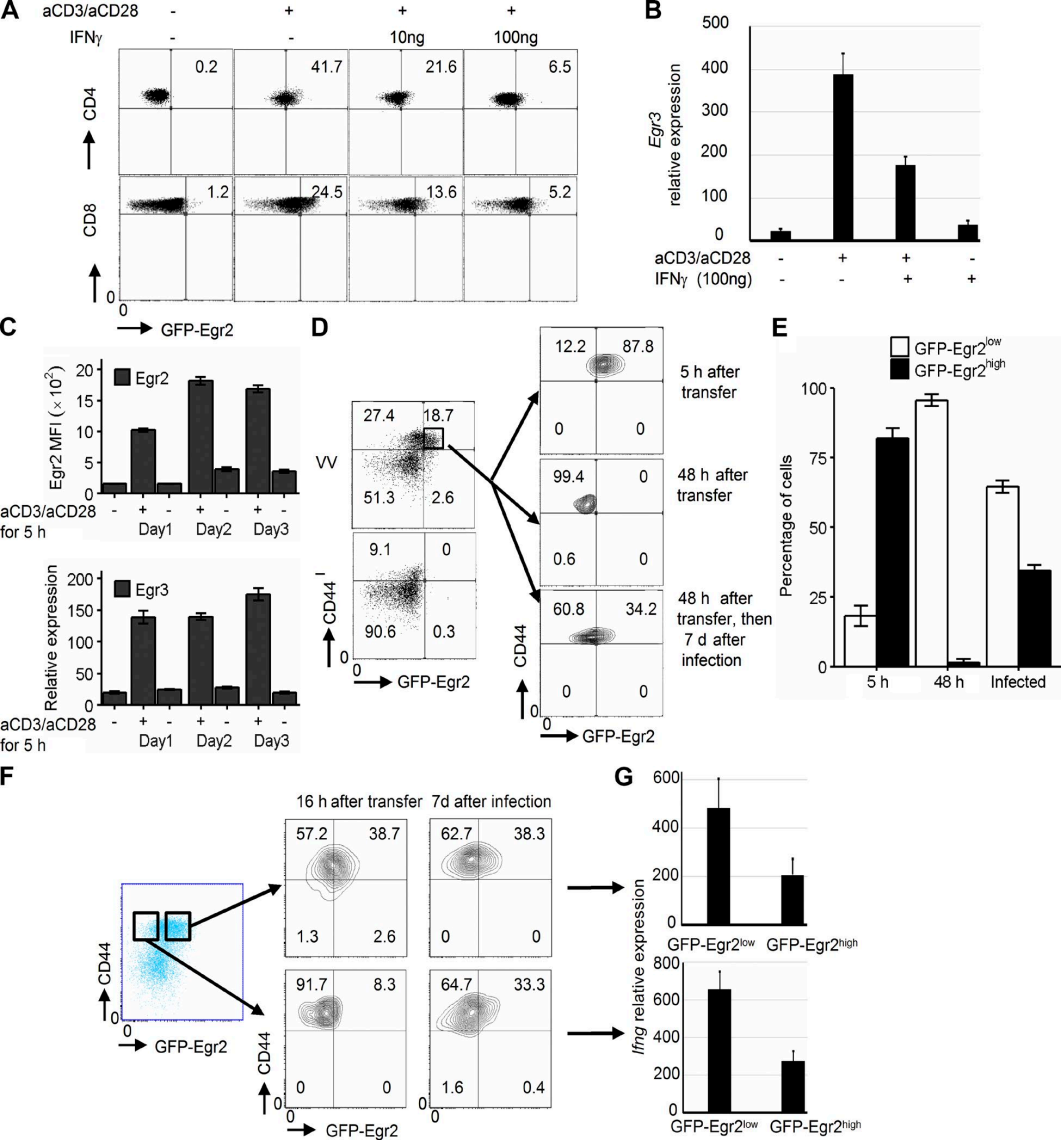
**Egr2 and 3 expression is repeatedly temporally regulated by TCR signaling in activated T cells.** (A and B) Naive CD4 and CD8 T cells were stimulated with the indicated stimuli for 16 h before analysis of GFP-Egr2 expression (A) or Egr3 expression by RT-PCR (B). (C) Naive CD4 T cells were repeatedly stimulated with 2 µg/ml anti-CD3 and 2 µg/ml CD28 for 5 h with 19-h intervals over a 3-d period. Egr2 MFI was analyzed by flow cytometry, and Egr3 expression was analyzed by RT-PCR, before and after each interval. (D and E) CD44^high^GFP-Egr2^high^CD45.2^+^ CD8 T cells from GFP-Egr2 knock-in mice 7 d after infection were sorted and adoptively transferred into CD45.1^+^ WT mice. Recipient mice were infected with OVA-VV_WR_ i.n. 48 h after transfer. Recipients were analyzed for Egr2 expression 5 or 48 h after transfer or 7 d after viral infection, as indicated. (F and G) CD44^high^GFP-Egr2^high^CD8^+^ and CD44^high^GFP-Egr2^low^CD8^+^ cells were isolated from CD45.2^+^GFP-Egr2 knock-in mice after infection with OVA-VV_WR_ for 7 d and then adoptively transferred into CD45.1^+^ WT mice. (F) GFP-Egr2 expression was examined 16 h after transfer or 7 d after OVA-VV_WR_ infection. (G) IFNγ expression by CD44^high^GFP-Egr2^high^CD8^+^ cells and CD44^high^GFP-Egr2^low^CD8^+^ cells isolated from infected recipient mice was analyzed by RT-PCR. Data in B and G are means ± SD, and data in C and E are means ± SEM. Data in A–C are representative of three independent experiments. Data in D–G are from pooled cells from four to five mice in each group and are representative of three independent experiments.

The defects in differentiation of T cells from CD2-Egr2 transgenic mice indicates that regulation of Egr2 and 3 expression is critical for optimal coupling of clonal expansion with differentiation of effector T cells. Analysis of Egr2 and 3 expression in naive T cells demonstrated that Egr2 and 3 were effectively induced by TCR stimulation, whereas their expression was suppressed by IFNγ (Fig. 9, A and B), suggesting a feedback mechanism to counter the inhibitory effect of Egr2 and 3 on effector differentiation.

These findings suggest the intriguing possibility that Egr2 and 3 expression in T cells during the course of an immune response may be temporally and repeatedly regulated based on changes in the microenvironment to adjust the level of expansion and differentiation. To assess this, we first kinetically analyzed Egr2 and 3 expression in naive CD4 T cells after repeated 5-h TCR stimulation at 19-h intervals. Egr2 and 3 were induced rapidly by initial stimulation (Fig. 9 C). After each interval, i.e., in the absence of TCR stimulation, Egr2 and 3 expression was completely abolished (Fig. 9 C). These results demonstrate that Egr2 and 3 expression in T cells is dependent on antigen stimulation.

Next we assessed regulation of Egr2 expression in vivo in response to viral infection. CD45.2^+^GFP-Egr2^high^CD44^high^CD8^+^ cells were isolated from GFP-Egr2 mice 7 d after infection with OVA-VV_WR_ and adoptively transferred into congenic CD45.1^+^ naive WT mice. The expression of Egr2 was assessed in recipients 5 and 48 h after transfer. Most Egr2^high^ cells maintained their expression after 5 h, but all the cells became GFP-Egr2^low^ after 48 h (Fig. 9, D and E). 48 h after adoptive transfer, the remaining recipient mice were infected with OVA-VV_WR_, and Egr2-expressing T cells among transferred CD8 cells were analyzed 7 d after infection. The results demonstrated that Egr2 was reinduced during infection of recipient mice (Fig. 9, D and E). Notably, the percentage of CD8 cells expressing high levels of Egr2 was similar to that observed in donor mice under the same infection conditions, indicating that Egr2 can be repeatedly induced after repeated exposure to antigens and that the level of each induction is based on the conditions in the microenvironment, not the expression level induced during previous induction. To further assess the temporal expression of Egr2 during viral infection, we isolated CD45.2^+^GFP-Egr2^high^CD44^high^CD8^+^ and CD45.2^+^GFP-Egr2^low^CD44^high^CD8^+^ cells and adoptively transferred them to separate CD45.1^+^ naive mice. 16 h after transfer, GFP-Egr2^low^ cells remained GFP-Egr2^low^, whereas GFP-Egr2^high^ were still largely GFP-Egr2^high^ in recipient mice (Fig. 9 F). However, 7 d after infection of recipient mice, the percentage of T cells expressing high levels of GFP-Egr2 was similar in both groups (Fig. 9 F). In each of these groups, the expression of IFNγ was higher in GFP-Egr2^low^ cells than in GFP-Egr2^high^ cells (Fig. 9 G), consistent with the results from infection of GFP-Egr2 mice (Fig. 5 I). Thus, the expansion and effector differentiation of individual T cells can be adjusted by temporal Egr2 and 3 expression in response to changes in the infected microenvironment to optimize responses until the pathogens have been eliminated.

## Discussion

The expansion and differentiation of T cells in response to infection are highly regulated by external signals, including inflammatory stimuli and the levels of antigens and cytokines (Kaech et al., 2002; Williams and Bevan, 2007; Kaech and Cui, 2012), to achieve optimal adaptive immune responses (Stemberger et al., 2007; King et al., 2012; Tubo et al., 2013). We have now found that Egr2 and 3, induced in response to antigen stimulation, are required for clonal expansion while potently inhibiting the differentiation of effector T cells. This temporal uncoupling of expansion and differentiation, mediated by Egr2 and 3, is essential for sufficient expansion of responding T cells and the control of pathology. We found that Egr2 and 3 expression is intricately regulated in a reciprocal fashion by antigen and inflammatory cytokines. The importance of proper regulation of Egr2 and 3 expression for adjusting expansion and differentiation is demonstrated by the reciprocal impairments in CD2-Egr2/3^−/−^ and CD2-Egr2 T cells. The failure to clear the virus in both CD2-Egr2/3^−/−^ and CD2-Egr2 transgenic mice, despite excessive effector differentiation in the former and enhanced proliferation in the latter, demonstrates the importance of optimal coupling of expansion and differentiation of T cells in response to viral infection.

Egr2 and 3 have an overlapping function in the regulation of the homeostasis of B and T cells (Li et al., 2012). Knockdown of Egr2 or Egr3 in established T cell lines renders T cells resistant to NFAT-mediated anergy in vitro (Harris et al., 2004), whereas Egr2 has been reported to regulate the function of Lag3-Treg cells (Okamura et al., 2009), indicating that Egr2 and 3 have important roles in T cell homeostasis. In contrast to their reported role in anergic T cells, we have demonstrated that Egr2 and 3 are required for clonal expansion of naive T cells in antiviral responses. Collectively, these data indicate that the function of Egr2 and 3 in regulation of clonal expansion differs from their roles in NFAT-mediated anergy and Lag3-Treg cells (Harris et al., 2004; Okamura et al., 2009), demonstrating that distinct mechanisms regulate T cells under homeostatic conditions and during viral infection.

The novel function of Egr2 and 3 in reciprocal regulation of proliferation and differentiation of naive T cells in adaptive immune responses is further extended by the intricate and reciprocal regulation of Egr2 and 3 expression by antigen and IFNγ. Reinduction of Egr2 and 3 after reencounter with antigen in vivo indicates that T cells can constantly adjust the extent of their proliferation and differentiation during the course of an infection, according to the levels of antigens and inflammation. This intricate regulatory mechanism, whereby antigen and IFNγ regulate Egr2 and 3 expression, together with the function of Egr2 and 3 in controlling proliferation and differentiation partly explains the profound diversity of individual T cells during an adaptive immune response (Stemberger et al., 2007; King et al., 2012; Buchholz et al., 2013; Gerlach et al., 2013; Tubo et al., 2013).

Transcriptional profiling demonstrated that Egr2 and 3 are upstream regulators of genes required for expansion and suppression of effector differentiation in both CD4 and CD8 T cells in response to viral infection. Among the proliferation regulators, Egr2 and 3 directly control the expression of *Myc* in both CD4 and CD8 T cells in response to viral infection, which is a key transcription factor for T cell proliferation (Wang et al., 2011; Nie et al., 2012). In addition, Egr2 and 3 also control several other proliferation regulators that are important for T cell proliferation such as *Myb* and *Bcl9* (Lieu et al., 2004; Mani et al., 2009). Together with our previous findings that Egr2 can enhance AP1 activation mediated by mitogenic TCR signaling in vitro (Li et al., 2012), these data demonstrate that Egr2 and 3 are essential for clonal expansion of T cells in adaptive immune responses.

The unprecedented finding of uncoupling of clonal expansion and effector differentiation of Egr2 expressing CD4 and CD8 T cells is associated with reciprocal regulation of repressors and promoters of effector differentiation by Egr2 and 3. In Egr2- and Egr3-deficient T cells, lineage-specific transcription factors (*Tbx21*, *Zeb2*, *Prdm1*, *Bhlhe40*, *Rora*, *Rorc*, *Runx2*, and *Runx3*) for differentiation of cytotoxic CD8, Th1, and Th17 cells (Kaech and Cui, 2012; Lin et al., 2014; Dominguez et al., 2015; Omilusik et al., 2015) were highly expressed, whereas repressors (*Id3* and *Bcl6*) of effector differentiation (Shaffer et al., 2000; Yang et al., 2011) were defective. Among these altered genes, Egr2 directly bound to the *Myc*, *Myb*, *Id3*, *Bcl6*, *Bhlhe40*, *Rora*, *Rorc*, and *Zeb2* loci, placing the function of Egr2 and 3 upstream of a network of transcription factors regulating proliferation and differentiation.

The transcriptome analysis not only reveals Egr2- and Egr3-mediated pathways in T cells, but also identifies the stages of adaptive immune responses at which they act. A comparison with the transcriptional profiles of TCR transgenic naive CD8 T cells analyzed at different stages of an immune response (Best et al., 2013), revealed that Egr2 and 3 induce expression of genes mainly involved in preparation for proliferation at the early stages of infection while repressing genes required for effector differentiation. The distinctive representation of down-regulated and up-regulated genes from Egr2- and Egr3-deficient T cells into early proliferation and late differentiation gene clusters, respectively, demonstrates that Egr2 and 3 expression defines a subset of cells that are about to proliferate after recent antigen encounter and that such cells are constantly being generated throughout the course of an infection.

T-bet is highly expressed in Egr2- and Egr3-deficient T cells in response to viral infection, which is in direct contrast to a recent report that Egr2 is required for T-bet expression and that Egr2 deficiency leads to defects in both T cell activation and effector differentiation (Du et al., 2014). Although we could not explain the reason for the conflicting findings, we conclude that T-bet is not defective in Egr2- and Egr3-deficient T cells.

Remarkably, despite suppressing effector differentiation, Egr2 directly bound and promoted the expression of Tfh-related transcription factors (*Bcl6*, *Lef1*, *Tcf7*, and *Ascl2*), whereas the Tfh repressor *Prdm1* was highly induced in Egr2- and Egr3-deficient CD4 T cells. These data are consistent with and extend our previous findings of Egr2- and Egr3-mediated Tfh cell differentiation (Ogbe et al., 2015). Recently, it has been suggested that Tfh is an early state of effector differentiation (Nakayamada et al., 2011), which further supports an upstream function for Egr2 and 3 in programming the early phase of T cell adaptive immune responses.

Egr2 and 3 are transiently expressed in naive T cells in response to acute viral infection, whereas the expression is sustained in chronic infection and tolerogenic conditions (Harris et al., 2004; Best et al., 2013; Stelekati et al., 2014). The sustained expression of Egr2 in T cells in lymphocytic choriomeningitis virus chronic infection is associated with defective effector function such as impaired production of IFNγ (Stelekati et al., 2014), suggesting that sustained expression of Egr2 and/or 3 caused by antigen persistence may be one of the mechanisms responsible for functional impairment of T cells in chronic infection.

Thus, regulation of the responses of individual T cells by Egr2 and 3, which themselves are regulated by the levels of antigens and inflammation, provides a flexible, individual, and intrinsic regulatory mechanism that determines the efficacy and pathology of an adaptive immune response. This novel function of Egr2 and 3, temporally induced by antigen while repressed by effector cytokines, is important for both optimal adaptive immune responses and the control of immunopathology.

## Materials and methods

### Mice

Generation of the GFP-Egr2 allele is described in Fig. 5 and the section below. GFP-Egr2 mice were backcrossed to C57BL/6 >10 times. GFP-Egr2 homozygous mice were used in this study. CD2-Egr2^−/−^ (CD2-Cre/Egr2^loxp/loxp^), Egr3^−/−^, CD2-Egr2/3^−/−^ (CD2-Cre/Egr2^loxp/loxp^/Egr3^−/−^), and CD2-Egr2 transgenic mice were reported previously (Tourtellotte and Milbrandt, 1998; Zhu et al., 2008; Li et al., 2011, 2012) and were backcrossed to C57BL/6 more than 30 times. C57BL/6 mice purchased from Charles River were used as controls in all experiments. All mice analyzed were 7–8 wk of age. No animal was excluded from the analysis, and the number of mice used was consistent with previous experiments using similar experimental designs. All mice were maintained in the Biological Services Unit, Brunel University, and used according to established institutional guidelines under the authority of a UK Home Office project license.

### Antibodies and flow cytometry

FITC-conjugated antibodies to B220 (11-0452-81, clone RA3-6B2), CD4 (11-0041-81, clone GK1.5), CD8 (11-0081-81, clone 53-6.7), CD45.1 (11-0453-81, clone A20), IFNγ (11-7311-81, clone XMG1.2), and TCRβ (11-5961-81, clone H57-597); PE-conjugated antibodies to CD3 (12-0031-81, clone 145-2C11), CD4 (12-0041-81, clone GK1.5), CD8 (12-0081-81, clone 53-6.7), CD25 (12-0251-81, clone PC61.5), CD62L (12-0621-81, clone MEL-14), CD69 (12-0691-81, clone H1.2F3), Ki-67 (12-5698-82, clone SolA15), Egr2 (12-6691-80, clone erongr2), and granzyme B (12-8898-80, clone NGZB); PerCP-labeled antibody to CD3 (45-0031-80, clone 145-2C11) and CD45.2 (45-0454-80, clone 104); allophycocyanin (APC)-conjugated antibodies to CD44 (17-0441-81, clone IM7), CD3 (17-0031-81, clone 145-2C11), TCRβ (17-5961-81, clone H57-597), Egr2 (17-6691-82, clone erongr2), and TNF (17-7321-81, clone MP6-XT22); and PEcy7-conjugated antibody to CD44 (25-0441-81, clone IM7) were obtained from eBioscience. PE-labeled anti-mouse TCR Vβ5.1, 5.2 (139504, clone MR9-4) was purchased from BioLegend. Antibody to CD3 (557306, clone 145-2C11) and CD28 (557393, clone 37.51) for stimulation and 7AAD were obtained from BD. FITC- and APC-labeled MHC/peptide tetramers consisting of H2Kb MHC molecules bearing OVA 257-264 SIINFEKL peptides were obtained from the NIH Tetramer Core facility (Emory University, Atlanta, GA). For flow cytometry analysis, single-cell suspensions were analyzed on a LSRII or Canto (BD), and the data were analyzed using FlowJo (Tree Star). Cell sorting was performed on a FACSAria sorter with DIVA option (BD).

### Cell isolation and stimulation

Naive CD4^+^ T cells were purified by negative selection using a MACS system (Miltenyi Biotec) or isolated by sorting CD25^−^CD4^+^CD44^high^CD62L^−^ and CD25^−^CD4^+^CD44^low^CD62L^+^ T cells by FACS after staining with FITC-conjugated CD4, PE-conjugated CD62L, PEcy7-conjugated CD44, and APC-conjugated CD25 antibodies. Cells were gated on CD25^−^ cells to exclude activated cells and Tregs. CD4^+^CD44^high^CD62L^−^GFP-Egr2^high^, CD4^+^CD44^high^CD62L^−^GFP-Egr2^low^, CD8^+^CD44^high^CD62L^−^GFP-Egr2^high^, and CD8^+^CD44^high^CD62L^−^GFP-Egr2^low^ T cells from virus-infected mice were sorted by FACS. CD8^+^ T cells from infected mice were isolated by antibody-coated beads using MACS. Purified CD4^+^ T cells were stimulated with plate-bound anti-CD3 at 5 µg/ml or the indicated concentrations and anti-CD28 antibodies (2 µg/ml) for the indicated times. Recombinant murine IFNγ (R&D Systems) was added where indicated.

CD8 T cells were stimulated in vitro with LB27.4 cells, which are B cell hybridoma cells that express both MHC class I and II proteins of the H-2 b haplotype (Antoniou and Watts, 2002). These cells were maintained in DMEM supplemented with 10% FCS, 2 mM l-glutamine, and antibiotics. Cells were routinely tested for mycoplasma contamination. Cells were infected with OVA-VV_WR_ at a multiplicity of infection of ∼5 and harvested between 8 and 12 h after infection. Cells were then resuspended in complete RPMI 1640 and used to stimulate CD8 cells at a ratio of 0.5:1 LB27.4/T cells unless otherwise stated.

For analysis of Egr2 expression, the cells were processed using the Foxp3 staining kit (eBioscience). For analysis of cytokine-producing cells, the cells were stimulated with 50 ng/ml PMA and 200 ng/ml ionomycin in the presence of Golgistop (BD) for 3 h before analysis of cytokine-producing cells by flow cytometry.

### Proliferation

GFP-Egr2–transduced cells were labeled with CellTrace Far-Red reagent (Invitrogen). The cells were stimulated for 72 h with 5 µg/ml anti-CD3 and 2 µg/ml anti-CD28 before analysis by flow cytometry.

Purified CD8 cells (5 × 10^4^) were incubated with OVA-VV_WR_–infected LB27.4 cells at 1:1 or 1:5 ratios in 96-well plates in triplicate for 48 h. A total of 1 µCi of [^3^H]TdR was added for the last 8 h of culture, and the cells were then harvested and subjected to scintillation counting to measure [^3^H]TdR incorporation.

### Generation of constructs

All PCR cloning steps were performed with Pfx polymerase, and all constructs were confirmed by sequencing.

### GFP-Egr2 knock-in construct

A BAC clone (MGC mouse RP23-88D4) containing the *Egr2* gene was obtained from Source Bioscience. The *Egr2*-targeting construct was cloned from this BAC clone in two parts: a 5′ BamHI fragment and a 3′ BglII fragment (Fig. 5 A). The 5′ fragment of the *Egr2* gene (3 kb) was cloned by PCR with the following primers: sense 5′-AATCGCGGATCCTCGCTGCTCC-3′ and antisense 5′-AATCGCGGATCCTGGTAGAGATCTCCT-3′; digested with BamHI; and inserted into a pBlueScript vector. The resulting construct was modified by site-directed mutagenesis to insert an NdeI site just after the start codon of the *Egr2* gene via PCR with the following primers: sense 5′-AGGTTGTGCGAGGAGCAAATGATGCATATGGTGAGCAAGGGCGA-3′ and antisense 5′-TCGCCCTTGCTCACCATATGCATCATTTGCTCCTCGCACAACCT-3′. The CDS of GFP was cloned by PCR to contain NdeI sites with the following primers: sense 5′-AATGATGCATATGGTGAGCAAGGGCGAGG-3′ and antisense 5′-TTGGCGGTCATATGATCTGAGTACTTGTACAGC-3′, and inserted into the Egr2 pBlueScript construct. The 3′ fragment of Egr2 (7 kb) was cloned by PCR with the following primers: sense 5′-AATCGCAGATCTCTACCAGGATCCTTCAGC-3′ and antisense 5′-AATCGCAGATCTGTCGTTTAAACAAATTTTTTTGTTGCTATTGG-3′; digested with BglII; and inserted into a pBlueScript vector. To enable positive selection after transfection, the Neomycin cassette was inserted at a NheI site downstream of the Egr2 ORF. The 3′ fragment of Egr2 with Neomycin was inserted into the BglII site on the pBlueScript construct containing the 5′ fragment of Egr2. To enable negative selection after transfection, the herpes simplex virus thymidine kinase (TK) cassette was inserted at a SacII site on the pBlueScript vector downstream of the 3′ fragment of Egr2. The plasmid was linearized with PciI for transfection of C57BL JM8ES cells. After karyotyping, two positive clones were used for blastocyst injection by Genetic Manipulation Services, Francis Crick Institute (Herts, UK). Chimeras were backcrossed more than 10 times to C57BL/6, and germline transmission was confirmed by genotyping with primers P1, 5′-TTGGGCGTTTGAAGTAATGG-3′ (long product sense, outside targeting construct); P2, 5′-GCTCAGTTCAACCCCTCTCC-3′ (short product sense); and P3, 5′-GGATTTTGTCTACGGCCTTG-3′ (common antisense). Expression of GFP-Egr2 in T cells was confirmed by flow cytometry and immunoblotting with both anti-GFP and anti-Egr2 antibodies.

### Quantitative real-time PCR

Total RNA was extracted from cells using TRIzol (Invitrogen) and reverse transcribed using random primers (Invitrogen). Quantitative real-time PCR was performed on a Rotor-Gene system (Corbett Robotics) using SYBR green PCR master mix (QIAGEN). The primers used are as follows: *Egr2*, sense 5′-CTTCAGCCGAAGTGACCACC-3′ and antisense 5′-GCTCTTCCGTTCCTTCTGCC-3′; *Egr3*, sense 5′-GCTCTTCCGTTCCTTCTGCC-3′ and antisense 5′-CGGTGTGAAAGGGTGGAAAT-3′; *Il2*, sense 5′-GCATGTTCTGGATTTGACTC-3′ and antisense 5′-CAGTTGCTGACTCATCATCG-3′; *Ifng*, sense 5′-CCATCAGCAACAACATAAGC-3′ and antisense 5′-AGCTCATTGAATGCTTGGCG-3′; *Gzmb*, sense 5′-GACCCAAAGACCAAACGTGC-3′ and antisense 5′-TGAAAGCACGTGGAGGTGAA-3′; *Ccr5*, sense 5′-GGAGGTGAGACATCCGTTCC-3′ and antisense 5′-GAATACCAGGGAGTAGAGTGGG-3′; *Id3*, sense 5′-ACATGAACCACTGCTACTCGC-3′ and antisense 5′-TGAGCTCAGCTGTCTGGATCG-3′; *Myc*, sense 5′-GTACCTCGTCCGATTCCACG-3′ and antisense 5′-GCTCTTCTTCAGAGTCGCTGC-3′; *Myb*, sense 5′-CTGAAGATGCTACCTCAGACCC-3′ and antisense 5′-TCCCGATTTCTCAGTTGGCG-3′; *Prdm1*, sense 5′-AACCTGAAGGTCCACCTGAG -3′ and antisense 5′-TGCTAAATCTCTTGTGGCAGAC-3′; *Tcf7*, sense 5′-CCCAGCTTTCTCCACTCTACG-3′ and antisense 5′-CTGTGAACTCCTTGCTTCTGGC-3′; *Tbx21*, sense 5′-CATTGCAGTGACTGCCTACC-3′ and antisense 5′-CACTCGTATCAACAGATGCG-3′; and *Gapdh*, sense 5′-TGCACCACCAACTGCTTAGC-3′ and antisense 5′-GGCATGGACTGTGGTCATGAG-3′.

The data were analyzed using the Rotor-Gene software. All samples were run in triplicate, and relative mRNA expression levels were obtained by normalizing against the level of *Gapdh* from the same sample under the same program according to relative expression = 2^(CTgapdh − CTtarget)^.

### Histological analysis

Tissues were fixed with 10% formalin in PBS and embedded in paraffin. Sections were stained with hematoxylin and eosin (H&E). Histological examination of tissue sections was done in a blinded manner.

### RNA-seq analysis

RNA was isolated and purified using TRIzol reagent (Thermo Fisher Scientific). RNA concentration and integrity were measured on a 2100 Bioanalyzer (Agilent Technologies). Only RNA samples with RNA integrity values >8.0 were considered for subsequent analysis. mRNA from T cells from independent experiments was processed for directional mRNA-seq library construction using the TruSeq Stranded mRNA Library Prep kit (Illumina) according to the manufacturer’s protocol. We performed 43-nt paired-end sequencing using an Illumina NextSeq platform and obtained ∼40 million reads per sample. Base calls, demultiplexing, and adapter trimming were performed with Illumina software. The short sequenced reads were mapped to the mm10 build of the mouse reference genome using the spliced aligner Tophat (Kim et al., 2013). Intermediate processing steps to remove secondary alignments and pairs where only one read was mapped were performed using Samtools (Li et al., 2009). We used several R/Bioconductor (R Core Team, 2015) packages to identify genes differentially expressed between GFP-Egr2 and Egr2/3^−/−^ T cells. In brief, numbers of reads mapped to each gene on the basis of the refGene UCSC database were counted, reported, and annotated using the BiocParallel, Rsamtools, GenomicAlignments, GenomicFeatures, and org.Mm.eg.db packages (Lawrence et al., 2013; Carlson, 2015; Morgan et al., 2015). Raw datasets have been submitted to the ArrayExpress database under accession no. E-MTAB-5338. To identify genes differentially expressed between groups, we used the R/Bioconductor package DESeq2 (Love et al., 2014) with default parameters. In brief, count data were first normalized and dispersion estimated before a negative binomial model was fitted with significance assessed by a Wald test. Resulting p-values were adjusted for multiple testing using the Benjamini–Hochberg procedure. Genes with an adjusted p-value ≤0.05 and an absolute fold change ≥1.5 were considered differentially expressed. The Venn diagram was created using the VennDiagram package (Chen, 2015).

Individual genes were plotted after normalization by sample read depth. For heat maps, a variance stabilizing transformation from the DESeq2 and vsn packages (Huber et al., 2002; Love et al., 2014) was applied to the dataset, and differentially expressed genes identified were selected and row-centered by subtraction of the mean expression level for each gene before clustering with the Diana algorithm from the cluster package (Maechler et al., 2015) and visualization with the pheatmap package (Kolde, 2015).

For the comparison with the data from Best et al. (2013), the probeset affyIDs in each cluster were converted to Entrez ID and Gene symbol using the R/Bioconductor package biomaRt (Durinck et al., 2005, 2009). For the volcano plots, the data were modeled as ∼Cell type + Genotype to generate a single value for each gene. The resulting Benjamini–Hochberg corrected p-values and log2 fold changes for the total dataset were plotted using the ggplot2 and gridExtra packages (Wickham, 2009; Auguie, 2015) and then the genes in each cluster were overlaid as indicated.

For building the model of the Egr2-centric transcriptional network, the direct Egr2 target genes identified in this study, together with their selected target genes from the literature, were visualized using Cytoscape (Shannon et al., 2003).

### ChIP assays

ChIP assays were performed according to the protocol supplied with the kit (9003) from Cell Signaling Technology. In brief, 5 × 10^7^ CD4 cells from WT mice were stimulated with anti-CD3 and anti-CD28 for 16 h. The cells were then cross-linked with 1% formaldehyde for 10 min at room temperature. After quenching of formaldehyde with 125 mM glycine, chromatin was sheared by sonication with a Bioruptor Pico sonication system (Diagenode). The fragmented chromatin was ∼300–1,000 bp as analyzed on agarose gels. After preclearing, chromatin (500 µg) was subjected to immunoprecipitation with specific anti-Egr2 antibody (eBioscience), or Ig as negative control, at 4°C overnight. Immunocomplexes were recovered by incubation with blocked protein G beads. DNA was purified according to the kit and used as template for PCR amplification in triplicate, with specific primers flanking the EMSA confirmed Egr2 binding sites. The primers used are as follows: *Ascl2*, sense 5′-TACTCTACAGGCACCTCCAC-3′ and antisense 5′-AATTGTCATTGGCCAAACGG-3′; *Bhlhe40*, sense 5′-CAGCCTCTGGAGAAGGCTAAGG-3′ and antisense 5′-GTTGGTAACGTGGGCGAACC-3′; *Id3*, sense 5′-GAGCGGAGTTATCAGCTGGAGG-3′ and antisense 5′-GGGCTGGGTTAAGATCGAAGC-3′; *Lef1*, sense 5′-CCCCTAGTCCCTAATTCTCGCC-3′ and antisense 5′-TTGCCAGTCTTTCTTCAACCTCC-3′; *Myb*, sense 5′-TGTCTCTACCACCCACATTTCC-3′ and antisense 5′-GGTTTGCTGGGAATCAACATGAGG-3′; *Myc*, sense 5′-CAAATCCGAGAGCCACAACCC-3′ and antisense 5′-AGGATTGCAAAATGACTACAGCC-3′; *Rora*, sense 5′-CTGTGGGCTTAAACCATGTGTGC-3′ and antisense 5′-CCGTCAATAAATGCTTCCTGTGG-3′; *Rorc*, sense 5′-CTCCTGCTGCAATGATGACACG-3′ and antisense 5′-AAAGAAAACAAGCCGGGGATGGG-3′; *Tcf7*, sense 5′-CAACGCATGTGATCACCCACC-3′ and antisense 5′-TCCTGAAAGAAGAGGCGTCCG-3′; and *Zeb2*, sense 5′-TATCATGAGAAAGGCTGTGCCTGG-3′ and antisense 5′-ATGCCCAGCCTTCCAAGTTAAGG-3′. Data are expressed as the percentage of input DNA recovered.

### EMSA

The consensus probe for Egr2 (5′-TGTAGGGGCGGGGGCGGGGTTA-3′) was labeled with Cyanine5 (Sigma-Aldrich) and used in binding reactions with nuclear extracts from CD4 T cells stimulated with anti-CD3 and anti-CD28 for 16 h. For supershift reactions, anti-Egr2 (eBioscience) was added after 10 min of incubation. The samples were electrophoresed on 5% polyacrylamide gels in 0.5× TBE. The gels were scanned using a Typhoon 9400 imager (GE Healthcare). For competition assays, oligos containing the Egr2 binding sites from each gene locus identified using Mulan (Ovcharenko et al., 2005) were added into the reaction mixtures before incubation. Probes used for competition assays are as follows: *Ascl2* (chr7:142969294-142969318), 5′-CCCTGGCGGAGGAGGCGGGAGCCGG-3′; *Bhlhe40* (chr6:108660027-108660047), 5′-GCCAGGCGGGGGAGGAGGAAG-3′; *Id3* (chr4:136144859-136144882), 5′-CTTCTCTCCTCCCCCGCCCAGAAC-3′; *Lef1* (chr3:131109827-131109848), 5′-GGGGCTAGAGTGGGCGGCGGGA-3′; *Myb* (chr10:21159419-21159440), 5′-GGGCCCACGCCCACGCTCCTGC-3′; *Myc* (chr15:61983490-61983511), 5′-GGGGGTGAGGGGGCGGGGAAAG-3′; *Rora* (chr9:69243892-69243913), 5′-GCTTGTTGTGTGGGAGGTGAGC-3′; *Rorc* (chr3:94371792-94371812), 5′-GGAGGGAGTGGGCGAGTCACG-3′; *Tcf7* (chr11:52274904-52274926), 5′-GGACGTCCTCCCACTCGCCCCTG-3′; and *Zeb2* (chr2:44979396-44979420), 5′-CTGCCCTCCTCCACCCCCTCCCCTG-3′.

### Lentiviral transduction

The lentiviral construct for GFP-Egr2 was generated by first creating a GFP-Egr2 fusion by insertion of the Egr2 CDS into the pEGFP-C3 vector and then transferring the GFP-Egr2 fusion construct into the lentivirus transfer vector PRRL via the NdeI and XbaI sites. The construct was confirmed by sequencing. Lentivirus was produced by cotransfection of three plasmids, the transfer vector, envelope vector, and packaging vector, into HEK293 cells and concentrated by ultracentrifugation of the supernatant on the second day after transfection. Naive CD4 cells at 10^6^ per well in a 24-well plate coated with anti-CD3 and anti-CD28 were infected with concentrated lentivirus at a multiplicity of infection of 50–100 (∼10^5^–10^6^ transducing units/ng of p24) as previously described (Li et al., 2012). The infected cells were incubated at 37°C for 7 h with gentle shaking before addition of 1 ml medium. The cells were harvested after 24 h, and the GFP-negative and -positive cells were isolated by cell sorting and used for functional analysis.

### Viruses and infection

Stocks of vaccinia virus (strain Western Reserve) encoding OVA (OVA-VV_WR_) were grown using TK143 cells in T175 flasks and infected at a multiplicity of infection of 0.5. Cells were harvested at 72 h, and virus was isolated by rapidly freeze-thawing the cell pellet three times in 5 ml DMEM containing 10% FCS as previously described (Ogbe et al., 2015). Cell debris was removed by centrifugation. Clarified supernatant was frozen at −80°C as virus stock. OVA-VV_WR_ stocks were titrated using TK143 cells.

Mice were infected i.n. with 2 × 10^5^ PFU of OVA-VV_WR_ in 10 µl of physiological saline. The mice were weighed and observed for illness daily, as previously described (Sofra et al., 2009). In vivo replication of OVA-VV_WR_ was examined by plaque assay on lung tissue samples which were removed, weighed, and ground with a mortar and pestle. Serial 10-fold dilutions of clarified supernatants were used to infect subconfluent monolayers of TK143 cells in triplicate in 24-well plates. The cells were fixed with formalin 2 d after infection and stained with 2% crystal violet in 40% methanol, and plaques were counted under an inverted microscope (CK2; Olympus).

### BM chimeras and OT1 retrogenic mice

BM was collected from CD2-Egr2/Egr3^−/−^ (CD45.2^+^) or WT C57BL/6 (CD45.1^+^) mice. For each chimera, 20 × 10^6^ cells of a 1:1 mixture of CD2-Egr2/Egr3^−/−^ and C57BL/6 BM cells were transferred intravenously into lethally irradiated (two doses of 550 rad) WT C57BL/6 (CD45.1^+^) recipients. For OT1 retrogenic mice, the OTI-2A.pMIG II construct, a gift from D. Vignali (plasmid 52111; Addgene; Holst et al., 2006a,b) was transfected into Phoenix cells (Takara Bio Inc.) as described (Zhu et al., 2008). BM cells isolated from CD2-Egr2/Egr3^−/−^ and WT C57BL/6 mice were cultured with IL-3, IL-6, and stem cell factor (BioLegend) and transduced with retroviral supernatant from transfected Phoenix cells by spin transduction as described (Holst et al., 2006a; Bettini et al., 2013). The transduced cells were analyzed for expression of GFP by flow cytometry. If GFP expression was detected, the cells were transferred into lethally irradiated (two doses of 550 rad) WT C57BL/6 recipients as described (Holst et al., 2006a; Bettini et al., 2013). Recipient mice were allowed 8 wk for reconstitution.

### Adoptive transfer

GFP^+^CD8^+^CD44^low^ cells were isolated from WT and Egr2/3^−/−^ OT1 retrogenic mice by FACS, and expression of CD62L and the OT1 TCR was confirmed by staining with APC-labeled Kb-SIINFEKL-tetramer and anti-CD62L. 3 × 10^5^ to 5 × 10^5^ WT and Egr2/3^−/−^ retrogenic OT1 cells were adoptively transferred to separate naive C57BL/6 mice. 24 h after transfer, the recipient mice were infected i.n. with 2 × 10^5^ PFU of OVA-VV_WR_.

### Statistics

To analyze the statistical significance of differences between groups, two-tailed Mann–Whitney tests, using the R package coin (Hothorn et al., 2008), or Kruskal–Wallis tests followed by pairwise comparisons using Conover tests, as implemented in the R package PMCMR (Pohlert, 2014), with Benjamini–Hochberg correction for multiple comparisons were used as indicated. Student’s unpaired two-tailed *t* tests were used for in vitro experiments. Differences with a p-value <0.05 were considered significant.

### Online supplemental material

Dataset S1 lists the genes identified as differentially expressed between GFP-Egr2^high^ and CD2-Egr2/3^−/−^ cells. Table S1 contains information used to construct the model of the “Egr2 centric” gene expression network in Fig. 8 D. The supplemental materials are provided as Excel files.

## Supplementary Material

Dataset S1 (Excel file)

Table S1 (Excel file)
